# Social determinants of health and cardiovascular outcomes in patients with heart failure

**DOI:** 10.1111/eci.13843

**Published:** 2022-08-15

**Authors:** Nicklas Vinter, Ameenathul M. Fawzy, David Gent, Wern Yew Ding, Søren Paaske Johnsen, Lars Frost, Ludovic Trinquart, Gregory Y. H. Lip

**Affiliations:** ^1^ Diagnostic Centre University Clinic for Development of Innovative Patient Pathways, Silkeborg Regional Hospital Silkeborg Denmark; ^2^ Department of Clinical Medicine Aarhus University Aarhus Denmark; ^3^ Department of Clinical Medicine, Danish Center for Clinical Health Services Research Aalborg University Aalborg Denmark; ^4^ Liverpool Centre for Cardiovascular Science University of Liverpool and Liverpool Heart and Chest Hospital Liverpool UK; ^5^ Tufts Clinical and Translational Science Institute Tufts University Boston Massachusetts USA; ^6^ Institute for Clinical Research and Health Policy Studies Tufts Medical Center Boston Massachusetts USA; ^7^ Department of Biostatistics Boston University School of Public Health Boston Massachusetts USA; ^8^ Department of Clinical Medicine Aalborg University Aalborg Denmark

**Keywords:** atrial fibrillation, cardiovascular death, education, income, myocardial infarction, stroke

## Abstract

**Background:**

We examined the associations between family income and educational attainment with incident atrial fibrillation (AF), myocardial infarction (MI), stroke and cardiovascular (CV) death among patients with newly‐diagnosed heart failure (HF).

**Methods:**

In a nationwide Danish registry of HF patients diagnosed between 2008 and 2018, we established a cohort for each outcome. When examining AF, MI and stroke, respectively, patients with a history of these outcomes at diagnosis of HF were excluded. We used cause‐specific proportional hazard models to estimate hazard ratios for tertile groups of family income and three levels of educational attainment.

**Results:**

Among 27,947 AF‐free patients, we found no association between income or education and incident AF. Among 27,309 MI‐free patients, we found that lower income (hazard ratio 1.28 [95% CI 1.11–1.48] and 1.11 [0.96–1.28] for lower and medium vs. higher income) and education (1.23 [1.04–1.45] and 1.15 [0.97–1.36] for lower and medium vs. higher education) were associated with MI. Among 36,801 stroke‐free patients, lower income was associated with stroke (1.38 [1.23–1.56] and 1.27 [1.12–1.44] for lower and medium vs. higher income) but not education. Lower income (1.56 [1.46–1.67] and 1.32 [1.23–1.42] for lower and medium vs. higher income) and education (1.20 [1.11–1.29] and 1.07 [0.99–1.15] for lower and medium vs. higher education) were associated with CV death.

**Conclusions:**

In patients with newly‐diagnosed HF, lower family income was associated with higher rates of acute MI, stroke and cardiovascular death. Lower educational attainment was associated with higher rates of acute MI and cardiovascular death. There was no evidence of associations between income and education with incident AF.

## 1 INTRODUCTION

Social determinants of health (SDOH) are nonmedical factors that influence health outcomes and cause health inequity between and within countries.^1^, ^2^ Household income, educational attainment, employment status and neighbourhood socioeconomic are examples of SDOH that are well‐established risk factors for cardiovascular diseases.^3^ SDOH can change throughout one's life, and assessment at the time of a cardiovascular diagnosis may provide valuable insights into how socioeconomic position influences subsequent healthcare and prognosis.^4^, ^5^

Heart failure (HF) is a common condition encountered in clinical practice. The global age‐adjusted incidence of HF has levelled off in recent decades, but the prevalence is increasing,^6^ and approximately 64 million individuals had a diagnosis of HF in 2017.^7^ The 5‐year survival proportion is about 50% after HF, and identification of prognostic indicators that improve risk stratification is crucial to facilitate targeted care.^8^ However, SDOH in HF has received limited attention, and a recent scientific statement from the American Heart Association underlined the necessity to address SDOH in the care of patients with HF.^9^

Previous studies on SDOH in HF have focussed on mortality and hospitalizations and reported substantial inequalities.^10^, ^11^, ^12^ To our knowledge, no study has thus far examined the associations between SDOH and the risk of other cardiovascular events among patients with HF. Using household income and educational attainment to identify HF patients at high risk of cardiovascular complications may guide individualized interventions to improve the prognosis of HF patients. Accordingly, the objectives of this study were to examine the association between SDOH and cardiovascular outcomes in patients with HF.

## 2 METHODS

Reporting of the study conforms to broad EQUATOR guidelines.^13^

### 2.1 Data sources

The Danish Heart Failure Registry (DHFR) is a nationwide clinical quality database that includes inpatients and outpatients with incident HF.^14^ The objectives of the DHFR are to monitor and improve the quality of care for Danish patients with HF. Registration of HF patients in the DHFR is mandatory for all Danish hospitals. A cardiologist must diagnose or validate any patient before enrolment.^14^ The inclusion criteria of the registry include the first‐time diagnosis of HF according to diagnostic criteria from the National Society for Cardiology and European Society of Cardiology: HF symptoms and objective signs of HF, and/or a possible clinical improvement on HF treatment. Exclusion criteria include HF caused by uncorrectable structural heart disease, HF caused by valvular heart disease, HF caused by rapid heart rhythm (including AF), isolated right‐sided HF, HF diagnosed concurrently with a primary diagnosis of acute myocardial infarction (MI) or HF patients diagnosed and treated by a private practitioner of cardiology.^14^ The cardiologist identifies these conditions in the patient's medical records. The DHFR provided the source population for this study.

The Danish National Patient Registry contains prospectively registered information on all inpatients, and after 1995, all outpatients.^15^ Individual‐level information is available on admission and discharge, surgical procedures performed, primary diagnosis and secondary diagnoses at discharge. Coding of diagnoses followed the Danish version of the International Classification of Diseases 8th Revision (ICD‐8) before 1994 and the 10th revision (ICD‐10) from 1994 and onwards.

The Danish National Prescription Registry contains individual‐level data on all dispensed prescriptions since 1994.^16^ Coding of medications followed the Anatomical Therapeutic Chemical Classification System.

The Danish Register of Causes of Death has since 1970 covered all deaths in a computerized form.^17^ The classification of cause of death is based on a certificate from a postmortem examination. The classification follows the ICD‐10 codes.^17^

Statistics Denmark provided information on family income and the highest level of education.

The Danish Civil Registration System provided individual‐level information on sex, date of birth, vital statistics and migration.^18^ Assignment of a unique 10‐digit Civil Registration number to all Danish citizens enabled unambiguous linkages of data across registries.

### 2.2 Design and populations

We conducted a nationwide registry‐based cohort study among patients with newly‐diagnosed HF between 2008 and 2018. To follow the patients for each cardiovascular outcome of interest, we established outcome‐specific cohorts. In each cohort, we excluded patients with a history of the outcome at the diagnosis of HF using ICD‐8 and ICD‐10 codes (Table S1). Additionally, we excluded HF patients who had lived in Denmark for less than 5 years before the diagnosis of HF to ensure sufficient time for the registry‐based identification of the history of diseases. In all cohorts, the baseline was on the day of the diagnosis of HF. The follow‐up period was from 2008 up to and including 2018.

### 2.3 Social determinants of health

Exposures of interest included individual‐level information on family income and the highest level of achieved education. We examined the exposures separately. Family income comprised the yearly disposable equivalent income, which is a comparable measure that accounts for the number of family members living together and their ages. Statistics Denmark generated the family‐specific estimate by dividing the total family income by a weighted average number of people in the family. We categorized individuals according to the tertiles of family income (lower, medium and higher) based on the distribution of the study population.

We categorized education into lower, medium and higher educational attainment, respectively, which followed the International Standard Classification of Education (ISCED). Early childhood, primary education and lower secondary education (ISCED 0–2) formed the lower group. General upper secondary education and vocational upper secondary education (ISCED 3) formed the medium group. Short‐cycle tertiary, medium‐length tertiary, bachelor‐level education or equivalent, second‐cycle, master‐level or equivalent and PhD level (ISCED 5–8) formed the higher group. ISCED 4 does not exist in Denmark.

### 2.4 Cardiovascular outcomes

Outcomes of interest included AF, acute MI, any stroke and cardiovascular death. We selected these outcomes because of the increased incidence and severity among HF patients. In addition, these events are frequently included as components of major cardiovascular events (MACE) considered in clinical trials. Definition of diagnoses followed the ICD‐10 codes and included all primary or secondary hospital diagnoses, and inpatient and outpatient diagnoses (Table S1). We retrieved information on cardiovascular death using the registered underlying cause of death coded as ICD‐10 I00‐I99.

### 2.5 Covariates

We considered the age at HF diagnosis, sex, clinical data, lifestyle factors and comorbidities as potential confounders.

Clinical data included left ventricular ejection fraction (LVEF) and New York Heart Association (NYHA) classification. Definition of LVEF categories followed the current classification^19^: preserved ≥50%; mid‐range >40%–49% and reduced ≤40%. Patients underwent echocardiography up to 7 days after the diagnosis of HF. However, an older echocardiographic examination up to 6 months before HF could be considered valid. The cardiologist treating the patient assessed the need for a new echocardiographic examination. NYHA classes were as I, II and III/IV, and ascertainment of NYHA class was at the diagnosis of HF or up to 12 weeks after the diagnosis.

Lifestyle factors included high alcohol consumption and smoking, which were reported to the DHFR at the diagnosis of HF. The definition of high alcohol consumption was more than 14 drinks per week for women and 21 drinks per week for men until July 1, 2015. After that date, the registry applied a lower threshold of more than 7 drinks per week for women and 14 drinks per week for men, following the revised recommendations from the Danish Health Authority. Smoking status was categorized into current smoking, former smoking or never smoking.

Comorbidities and conditions included a history of MI, any stroke, AF, diabetes mellitus, chronic obstructive pulmonary disease, hypertension, chronic kidney disease, valvular heart disease and obesity (Table S2).

### 2.6 Statistical analyses

Time at risk began on the day of HF diagnosis. In each cohort, patients contributed to risk‐time until the date of the cardiovascular outcome of interest, death, heart transplantation, emigration or end of follow‐up, whichever came first.

Because we were interested in the etiological association between SDOH and each cardiovascular complication, rather than predicting who exactly will develop the complication before dying (or dying from noncardiovascular causes), we fitted cause‐specific proportional hazard models.^20^ It is equivalent to censoring the competing events (death or noncardiovascular death and heart transplantation) and fitting Cox models. We estimated cause‐specific hazard ratios (HR) with 95% confidence intervals (95% CI) for the associations. The first model was adjusted for age and sex (Model 1). We then fitted a model that adjusted for age, sex, clinical characteristics and comorbidities (Model 2). Finally, we fitted a model adjusted for age, sex, clinical characteristics, comorbidities and lifestyle factors (Model 3). We tested for the association between the exposure, income or education as a 3‐level covariate, and the cause‐specific hazard of the event of interest by using likelihood ratio tests comparing model 3 with and without the exposure. In addition, we plotted the multivariable‐adjusted smoothed hazard functions in each exposure group according to family income and educational attainment, and for each outcome, the hazard functions were estimated by kernel smooths of the estimated hazard contributions in model 3.

We tested for statistical interaction between income and education by including an interaction term in Model 3. We performed subgroup analyses by sex. We reported the sex‐specific associations and included an interaction term between exposure and sex in Model 3.

We assessed the proportional hazards assumption by using graphs of the Schoenfeld residuals and there was no evidence of departure from proportional hazards.

To account for missing values in weekly alcohol intake, smoking, LVEF or NYHA classification, we used multiple imputations by chained equations. The imputation models included all predictors, an event indicator and the Nelson–Aalen estimator of the cumulative hazard of the event of interest. We generated 10 datasets and combined the estimates from the datasets using Rubin's rules.^21^

Analyses were performed in stata (StataCorp. 2019: Release 16.1: StataCorp LLC).

### 2.7 Ethics

The Danish Health Data Authority, Statistics Denmark and the Danish Data Protection Agency approved this study. Registry‐based studies do not require approval from an ethics committee according to Danish law.

## 3 RESULTS

### 3.1 Baseline characteristics

**FIGURE 1:**
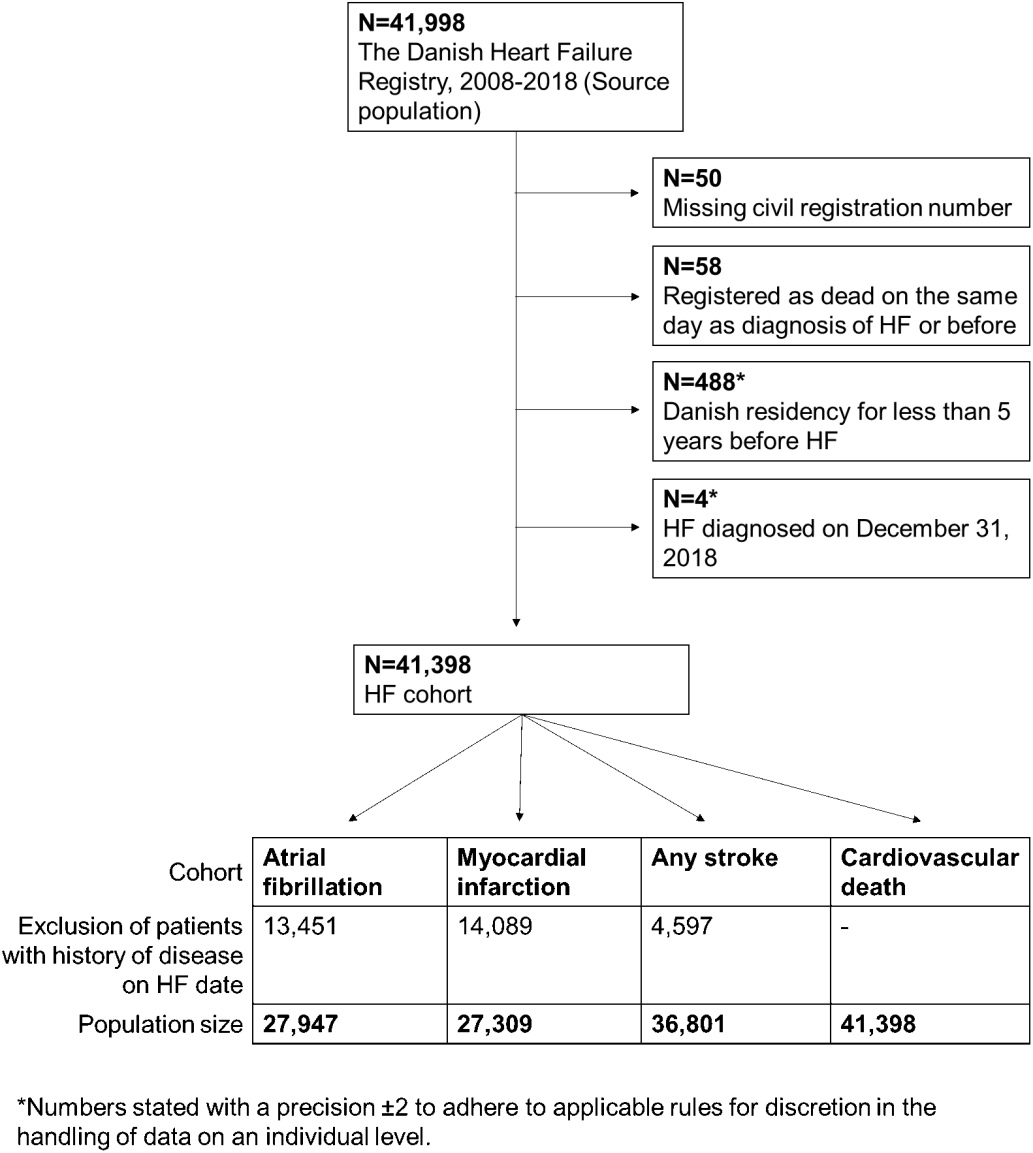
Flow diagram illustrating the selection of participants for each outcome of interest

Between 2008 and 2018, 41,398 patients were diagnosed with incident HF (Figure 1), 33% were female and the mean age was 70 years (Table 1). The cohort for incident AF included 27,947 patients (34% females and mean age 69 years), the cohort for incident MI included 27,309 patients (35% female and mean age 61 years) and the cohort for incident stroke included 36,801 patients (33% female and mean age 70 years). Tables S3–S13 show baseline characteristics and characteristics stratified by level of income and education. In relation to incident AF, MI, stroke and cardiovascular death, there was a decrease in the proportion of female patients and the mean age with higher income. In all cohorts, the mean age was highest and the proportion of female patients was highest among patients with lower education, but we noted no clear trends across all three groups of educational attainment.

**TABLE 1:** Baseline characteristics of all patients with HF

Characteristics	*N* = 41,398
Demographics	
Female sex, *N* (%)	13,456 (32.5)
Age, years, mean (SD)	70.2 (12.7)
Lifestyle factors	
Elevated alcohol consumption, *N* (%)	3336 (9.3)
Smoking status, *N* (%)	
Never	10,206 (27.3)
Former	16,641 (44.5)
Current	10,551 (28.2)
Clinical characteristics	
LVEF, *N* (%)	
<25%	9750 (24.2)
25%–40%	24,985 (62.0)
>40%–49%	2700 (6.7)
≥50%	2874 (7.1)
NYHA class, *N* (%)	
I	5492 (14.8)
II	22,295 (59.9)
III/IV	9409 (25.3)
Comorbidities and conditions	
Myocardial infarction, *N* (%)	14,089 (34.0)
Any stroke, *N* (%)	4597 (11.1)
Atrial fibrillation, *N* (%)	13,451 (32.5)
Diabetes mellitus, *N* (%)	8590 (20.8)
Chronic obstructive pulmonary disease, *N* (%)	5543 (13.4)
Hypertension, *N* (%)	17,774 (42.9)
Chronic kidney disease, *N* (%)	3358 (8.1)
Valvular disease, *N* (%)	4787 (11.6)
Obesity, *N* (%)	3572 (8.6)
Socioeconomic factors	
Family income, *N* (%)	
Lower (<49,606 euros)	15,173 (37.0)
Medium (49,608–68,011 euros)	13,317 (32.5)
Higher (>68,012 euros)	12,542 (30.6)
Highest completed education, *N* (%)	
Lower	17,365 (42.0)
Medium	15,939 (38.5)
Higher	6309 (15.2)

*Note:* Missing values (%) for alcohol: 5641 (13.6); smoking: 4000 (9.7); LVEF: 1089 (2.6); NYHA: 4202 (10.2); income: 366 (0.9); education 1785 (4.3).

Abbreviations: HF, heart failure; LVEF, left ventricular ejection fraction.

### 3.2 Risk of AF

The median follow‐up time was 5.7 years (Q1–Q3: 2.9–8.2) and the longest follow‐up was 11.0 years. During follow‐up, 4471 patients were diagnosed with incident AF and the corresponding incidence rate was 45.6 per 1000 person‐years. Figure S1 shows the multivariable‐adjusted smoothed hazard rates AF by levels of income and education, respectively. In multivariable‐adjusted cause‐specific Cox regression models, we found no evidence of association between family income and AF (lower vs. higher income: HR 0.98, 95% CI 0.91–1.06; medium vs. higher income: HR 0.97, 95% CI 0.90–1.05, *p* = .75) or educational attainment and AF (lower vs. higher education: HR 0.93, 95% CI 0.85–1.02; medium vs. higher education: HR 0.93, 95% CI 0.85–1.02; *p* = .28, Tables 2 and 3). Our analysis of interaction between income and education supported no statistical evidence (interaction test *p* = .20, Table S14).

**TABLE 2:** Cause‐specific hazard ratios with 95% confidence intervals for associations between family income and cardiovascular outcomes in HF patients

	Model 1	Model 2	Model 3	*p* Value in model 3
Atrial fibrillation				
Lower income	1.00 (0.93–1.08)	0.97 (0.90–1.05)	0.98 (0.91–1.06)	.75
Medium income	0.98 (0.91–1.06)	0.96 (0.89–1.04)	0.97 (0.90–1.05)	
Higher income	1.00 (ref)	1.00 (ref)	1.00 (ref)	
Myocardial infarction				
Lower income	1.44 (1.26–1.66)	1.32 (1.15–1.52)	1.28 (1.11–1.48)	.002
Medium income	1.21 (1.05–1.40)	1.13 (0.98–1.31)	1.11 (0.96–1.28)	
Higher income	1.00 (ref)	1.00 (ref)	1.00 (ref)	
Any stroke				
Lower income	1.49 (1.33–1.67)	1.41 (1.25–1.59)	1.38 (1.23–1.56)	<.001
Medium income	1.34 (1.19–1.51)	1.29 (1.14–1.45)	1.27 (1.12–1.44)	
Higher income	1.00 (ref)	1.00 (ref)	1.00 (ref)	
Cardiovascular death				
Lower income	1.76 (1.64–1.88)	1.59 (1.49–1.70)	1.56 (1.46–1.67)	<.001
Medium income	1.42 (1.32–1.52)	1.34 (1.24–1.44)	1.32 (1.23–1.42)	
Higher income	1.00 (ref)	1.00 (ref)	1.00 (ref)	

*Note:* Model 1: adjusted for age and sex. Model 2: Adjusted for all covariates listed in Table 1 except smoking and alcohol. Model 3: Adjusted for all covariates listed in Table 1.

Abbreviation: HF, heart failure.

**TABLE 3:** Cause‐specific hazard ratios with 95% confidence intervals for associations between educational attainment and cardiovascular outcomes in HF patients

	Model 1	Model 2	Model 3	*p* Value in model 3
Atrial fibrillation				
Lower education	0.94 (0.86–1.02)	0.92 (0.84–1.01)	0.93 (0.85–1.02)	.28
Medium education	0.93 (0.85–1.02)	0.93 (0.85–1.02)	0.93 (0.85–1.02)	
Higher education	1.00 (ref)	1.00 (ref)	1.00 (ref)	
Myocardial infarction				
Lower education	1.37 (1.16–1.63)	1.27 (1.07–1.50)	1.23 (1.04–1.45)	.06
Medium education	1.23 (1.04–1.47)	1.17 (0.98–1.39)	1.15 (0.97–1.36)	
Higher education	1.00 (ref)	1.00 (ref)	1.00 (ref)	
Any stroke				
Lower education	1.05 (0.92–1.20)	1.01 (0.88–1.15)	1.00 (0.87–1.14)	.93
Medium education	1.01 (0.88–1.15)	0.99 (0.86–1.13)	0.98 (0.86–1.12)	
Higher education	1.00 (ref)	1.00 (ref)	1.00 (ref)	
Cardiovascular death				
Lower education	1.31 (1.22–1.41)	1.21 (1.12–1.30)	1.20 (1.11–1.29)	<.001
Medium education	1.11 (1.03–1.20)	1.08 (1.00–1.16)	1.07 (0.99–1.15)	
Higher education	1.00 (ref)	1.00 (ref)	1.00 (ref)	

*Note:* Model 1: adjusted for age and sex. Model 2: Adjusted for all covariates listed in Table 1 except smoking and alcohol. Model 3: Adjusted for all covariates listed in Table 1.

Abbreviation: HF, heart failure.

The sex‐specific analysis indicated statistical evidence of interaction by sex (interaction test *p* = .005), which was driven by increasing rates of AF in women with lower family income (lower income: HR 1.11, 95% CI 0.95–1.30; medium income: HR 0.97, 95% CI 0.83–1.15; *p* < .09) but seemingly decreased rates of AF among men with lower income (lower income: HR 0.94, 95% CI 0.86–1.02; medium income: HR 0.98, 95% CI 0.89–1.07; *p* = .31), although there was no evidence of association in either subgroup (Table S15).

### 3.3 Risk of acute MI

During a median follow‐up of 5.7 years (Q1–Q3: 2.8–8.4), 1391 patients were diagnosed with acute MI. The longest follow‐up was 11.0 years. The incidence rate of acute MI was 13.9 per 1000 person‐years. In multivariable‐adjusted cause‐specific Cox regression models, we found that lower family income (lower income: HR 1.28, 95% CI 1.11–1.48; medium income: HR 1.11, 95% CI 0.96–1.28; *p* = .002) and lower educational attainment (lower education: HR 1.23, 95% CI 1.04–1.45; medium education: HR 1.15, 95% CI 0.97–1.36; *p* = .06) were associated with increased rates of MI (Tables 2 and 3 and Figure S1). Our analysis of interaction between income and education supported no statistical evidence (interaction test *p* = .95, Table S16). The sex‐specific analysis indicated no statistical evidence of interaction by sex (Table S17).

### 3.4 Risk of any stroke

During follow‐up, 2089 patients were diagnosed with any stroke and the corresponding incidence rate was 15.0 per 1000 person‐years. The median follow‐up time was 5.8 years (Q1–Q3: 3.0–8.4) and the longest follow‐up was 11.0 years. In multivariable‐adjusted cause‐specific Cox regression models, we found statistical evidence of an associations between income (lower income: HR 1.38, 95% CI 1.23–1.56; medium income: HR 1.27, 95% CI 1.12–1.44; *p* < .001) and incident stroke but not between education and incident stroke (Lower education: HR 1.00, 95% CI 0.87–1.14; medium education: HR 0.98, 95% CI 0.86–1.12, *p* = .93, Tables 2 and 3 and Figure S1). We found no statistical evidence of interaction between income and education (interaction test *p* = .998, Table S18) or by sex (interaction test *p* = .97 for income and *p* = .18 for education, Table S19).

### 3.5 Risk of cardiovascular death

The median follow‐up was 5.0 years (Q1–Q3: 2.4–7.7) and the longest follow‐up was 11.0 years. Cardiovascular disease was the registered underlying cause of death for 7528 patients. The corresponding cardiovascular mortality rate was 49.6 per 1000 person‐years. In multivariable‐adjusted cause‐specific Cox regression models, we found statistical evidence of an associations between income and cardiovascular death (lower income: HR 1.56, 95% CI 1.46–1.67; medium income: HR 1.32, 95% CI 1.23–1.54; *p* < .001), and education and cardiovascular death (lower education: HR 1.12, 95% CI 1.11–1.29; medium education: HR 1.07, 95% CI 0.99–1.15; *p* < .001, Tables 2 and 3 and Figure S1). We found no statistical evidence of interaction between income and education (interaction test *p* = .59, Table S20) or by sex (Table S21).

## 4 DISCUSSION

In this large nationwide cohort study of Danish patients with newly‐diagnosed HF, we found that lower income level was associated with a higher rate of incident MI, any stroke and cardiovascular death, respectively, after adjustment for cardiovascular risk factors at the time of the HF diagnosis. Lower level of educational attainment was associated with a higher rate of incident MI and cardiovascular death after adjustment for cardiovascular risk factors at the time of the HF diagnosis. We found no statistical evidence of interaction between income and education and no clinically significant differences by sex. All results originated from the strong Danish social welfare system, which is characterized by free access to healthcare and education. Consequently, the observed differences may be even more dominant in countries without similar social security.

Previous studies on SDOH in HF have demonstrated associations with mortality and hospitalizations.^10^, ^11^, ^12^ Our study extends the literature by showing that a lower socioeconomic position at HF diagnosis is associated with a higher risk of acute MI, stroke and cardiovascular death but is not associated with AF. Reasons for the association between stroke and income but not education remain unknown. In contrast to our findings, Witte et al.^12^ found no association between an Index of Multiple Deprivation and cardiovascular mortality among 1802 patients with HF included from four UK hospitals. There were two important methodological differences between the studies. First, Witte et al. included outpatients with stable clinical signs and symptoms of HF for 3 months and with an ejection fraction of ≤45%. Second, Witte et al. used Index of Multiple Deprivation as exposure, which is an area‐based index according to postal codes and not an individual‐based index.

We found no evidence of association with incident AF, and it suggests less inequality with similar absolute risks between patients at lower and higher socioeconomic positions. This finding is consistent with results from the Framingham Heart Study, which found no evidence of the association between education or household income and lifetime risk of AF.^22^ A possible explanation is that regular follow‐up of patients with HF is necessary to evaluate the clinical status including heart rhythm and ensure optimal care. The most recent European guidelines for HF recommend intervals of clinical follow‐up of no more than 6 months, and the follow‐ups should be more frequent among patients recently discharged.^23^ Regular follow‐up likely detects chronic events such as AF more frequently.

The results of this study demonstrate that other mechanisms than unequal access to healthcare may account for the association between socioeconomic position and cardiovascular events in HF. SODHs are linked to cardiovascular disease through multiple interrelated pathways. As suggested by a previous Danish paper on HF patients, lower education and income are associated with lower fulfilment of process performance measures of HF care that may be associated with an increased risk of poor clinical outcomes.^24^ As our analyses adjusted for several cardiovascular risk factors at the time of HF, the underlying mechanisms may manifest themselves after the diagnosis of HF and could be targeted for interventions such as optimization of the quality of HF treatment. Further studies are needed to examine mediating pathways and interventions to improve the prognosis.

Considering both income and education as markers of acute MI, any stroke and cardiovascular death in patients with newly‐diagnosed HF seems reasonable and may have implications. Systematic assessment of income and education in HF may guide the identification of high‐risk patients to assess resources and initiate interventions towards underlying causes of low socioeconomic position. However, no standardized screening tools have been implemented in HF thus far.^9^, ^23^ Possible interventions have been discussed in a scientific statement by the American Heart Association and may include access to healthcare, social support and education.^9^ However, a similar European initiative for HF is warranted. Finally, this study may facilitate the establishment of pragmatic trials to examine optimal methods for assessing SDOH and improving prognosis.

### 4.1 Limitations

We excluded patients with a registered history of outcomes and patients with unregistered information have not been excluded. For example, we may have included patients with undiagnosed AF because we did not evaluate the patients clinically. However, it is less likely that patients with a history of acute events, such as MI and stroke were missed. Patients from lower socioeconomic positions may stay away from diagnostic workups at the hospitals, and the patients will not be included in the Danish HF registry. Therefore, our effect estimates may be biased towards the null, but the number of missed eligible patients are unknown. Additionally, we had no data on patients treated in a specialized out‐of‐hospital cardiology practice, but the number of patients is expected to be low.

The National Patient Registry provided information on diagnoses. In general, if patients characterized by lower socioeconomic position avoid healthcare visits, differential misclassification is possible and the incidence of a given cardiovascular outcome of interest may be systematically underestimated in lower socioeconomic groups. However, misclassification of acute events is unlikely. We did not review electrocardiograms to validate the diagnosis of AF, coronary angiograms to validate MI or imaging to validate stroke. However, validation studies of the AF diagnosis in the Danish National Patient Registry have reported positive predictive values of 92% and 95%.^25^, ^26^ The positive predictive value of first‐time MI is 97% and 79% for stroke.^26^, ^27^ As the classification of cause of death originates from subjective assessment, the data may be prone to misclassification. However, we consider it unlikely that the misclassification should depend on socioeconomic position.

Our statistical models adjusted for many essential covariates; however, we cannot rule out residual or unaccounted confounding, and the included risk factors at baseline may not be perfectly representative of the cardiovascular risk. For instance, we had no information on diet and physical activity. Furthermore, information on smoking did not reflect accumulated exposure.

Finally, as the data for this study originated from a tax‐financed universal healthcare system, generalizability may be limited. Given the nature of the Danish healthcare system, it is unlikely that unequal access to healthcare services or education influenced the observed effects substantially. However, similar associations between SDOH and clinical outcomes in HF may not be evident in countries where most of the healthcare system is privately funded. Additionally, the inclusion and exclusion criteria of the DHFR may limit the generalizability to different HF cohorts.

## 5 CONCLUSION

Among patients with newly‐diagnosed HF, the status of socioeconomic determinants of health at the time of diagnosis of HF was associated with cardiovascular outcomes in a universal welfare system with free access to healthcare. Lower income was associated with higher risks of acute MI, stroke and cardiovascular death. Lower education was associated with higher risks of acute MI and cardiovascular death. Income and education were not associated with incident AF. The results underline the importance of targeting patients with HF and low socioeconomic positions for preventive strategies.

## CONFLICT OF INTEREST

LF: Supported by a grant from the Health Research Foundation of Central Denmark Region. Consultant for Pfizer and BMS. GYHL: Consultant and speaker for BMS/Pfizer, Boehringer Ingelheim and Daiichi‐Sankyo. Remaining authors declare no conflicts of interest. No fees are received personally.

## Supporting information


**Appendix S1** Data
