# Supplementary material for: Social determinants of health and cardiovascular outcomes in patients with heart failure
Source: Eur J Clin Invest. 2022 Aug 15;52(11):e13843. doi: 10.1111/eci.13843 (PMC9786545; doi:10.1111/eci.13843)
Supplement: Supplementary file 1 — Appendix S1 Data [file ECI-52-e13843-s001.docx]

**SUPPLEMENTAL MATERIAL**

**Social determinants of health and incident cardiovascular outcomes in patients with heart failure.**

**N. Vinter, AM. Fawzy, D. Gent, WY. Ding, SP. Johnsen, L. Frost, L. Trinquart, GYH. Lip**

[Methods 3](#_Toc98933750)

[Supplemental Table 1. Definition of outcomes. 3](#_Toc98933751)

[Supplemental Table 2. Definitions of covariates and data sources. 4](#_Toc98933752)

[Baseline characteristics of cohorts 7](#_Toc98933753)

[Supplemental Table 3. Baseline characteristics of patients with no history of AF 7](#_Toc98933754)

[Supplemental Table 4. Baseline characteristics of patients with no history of AF, by income 8](#_Toc98933755)

[Supplemental Table 5. Baseline characteristics of patients with no history of AF, by education 10](#_Toc98933756)

[Supplemental Table 6. Baseline characteristics of patients with no history of myocardial infarction. 12](#_Toc98933757)

[Supplemental Table 7. Baseline characteristics of patients with no history of myocardial infarction, by income 13](#_Toc98933758)

[Supplemental Table 8. Baseline characteristics of patients with no history of myocardial infarction, by education 15](#_Toc98933759)

[Supplemental Table 9. Baseline characteristics of patients with no history of any stroke. 17](#_Toc98933760)

[Supplemental Table 10. Baseline characteristics of patients with no history of any stroke, by income. 18](#_Toc98933761)

[Supplemental Table 11. Baseline characteristics of patients with no history of any stroke, by education 20](#_Toc98933762)

[Supplemental table 12. Baseline characteristics of all patients, by income. 22](#_Toc98933763)

[Supplemental Table 13. Baseline characteristics of all patients, by education 24](#_Toc98933764)

[Atrial fibrillation 26](#_Toc98933765)

[Supplemental Table 14. Hazard ratios with 95% CI for incident AF and interaction between income and education in the multivariable-adjusted* models. 26](#_Toc98933766)

[Supplement Table 15. Hazard ratios with 95% CI for the association between socioeconomic factors and incident AF, by sex. 27](#_Toc98933767)

[Myocardial infarction 28](#_Toc98933768)

[Supplemental Table 16. Hazard ratios with 95% CI for incident myocardial infarction and interaction between income and education in the multivariable-adjusted* models. 28](#_Toc98933769)

[Supplement Table 17. Hazard ratios with 95% CI for the association between socioeconomic factors and incident myocardial infarction, by sex. 29](#_Toc98933770)

[Any stroke 30](#_Toc98933771)

[Supplemental Table 18. Hazard ratios with 95% CI for incident stroke and interaction between income and education in the multivariable-adjusted* models. 30](#_Toc98933772)

[Supplement Table 19. Hazard ratios with 95% CI for the association between socioeconomic factors and incident stroke, by sex. 31](#_Toc98933773)

[Cardiovascular death 32](#_Toc98933774)

[Supplemental Table 20. Hazard ratios with 95% CI for cardiovascular death and interaction between income and education in the multivariable-adjusted* models. 32](#_Toc98933775)

[Supplement Table 21. Hazard ratios with 95% CI for the association between socioeconomic factors and cardiovascular death, by sex. 33](#_Toc98933776)

[Figures 34](#_Toc98933777)

[Supplemental Figure 1. Smoothed multivariable-adjusted hazard functions according to family income and education for each outcome of interest. 34](#_Toc98933778)

# Methods

## Supplemental Table 1. Definition of outcomes.

| **Diagnosis** | **ICD-8 code** | **ICD-10 code** | **Type of diagnosis** | **Type of patient** |
| --- | --- | --- | --- | --- |
| Atrial fibrillation (or atrial flutter) | 42793 42794 | I48 | Primary and secondary | Inpatient- and outpatient |
| Acute myocardial infarction | 410 | I21 I22 I23 | Primary and secondary | Inpatient- and outpatient |
| Any stroke | 430 431 433 434 | I60 I61 I62 I63 I64 | Primary and secondary | Inpatient- and outpatient |

##

## Supplemental Table 2. Definitions of covariates and data sources.

| **Characteristics** | **Primary source** | **Secondary source(s)** | **ICD-8** | **ICD-10** | **ATC** | **Procedure** | **Time window prior to index date** |
| --- | --- | --- | --- | --- | --- | --- | --- |
| Elevated alcohol consumption | DFHR | - | - | - | - | - | - |
| Smoking status | DFHR | - | - | - | - | - | - |
| LVEF | DFHR |  | - | - | - | - | - |
| NYHA class | DFHR | - | - | - | - | - | - |
| Myocardial infarction | DNPR | - | 410 | I21 I22 I23 | - | - | Ever |
| Ischemic stroke | DNPR | - | 433 434 | I63 I64 | - | - | Ever |
| Diabetes mellitus* | DNPR+DPR | - | 249 250 | E100 E101 E109 E110 E111 E119 | A10 | - | ICD: ever  ATC: 6 months |
| Chronic obstructive pulmonary disease | DNPR | - | - | J44 | - | - | Ever |
| Hypertension** | DFHR | DNPR+DPR | 400 401 402 403 404 | I10 I11 I12 I13 I15 | Classes of antihypertensive drugs:  *α adrenergic blockers:* C02A; C02B; C02C  *Non-loop diuretics:* C02DA; C02L; C03A; C03B; C03D; C03E; C03X; C07B; C07C; C07D; C08G; C09BA; C09DA; C09XA52  *Vasodilators:* C02DB; C02DD; C02DG; C04; C05  *β blockers:* C07  *Calcium channel blockers:* C08; C09BB; C09DB  *Renin-angiotensin system inhibitors:* C09 | - | ICD: Ever  ATC: 6 months |
| Chronic kidney disease | DNPR | - | - | E102 E112 E142 I120 I131 I132 I150 I151 N03 N05 N06 N07 N08 N110 N14 N15 N16 N18 N19 N26 N27 N280 N391 Q61 | - | - | Ever |
| Obesity | DNPR |  | 277 | E66 | - | - | Ever |
| Valvular heart disease | DNPR | - | 394-396, 4240, 4241 | I05-I08 I34-I37 Z952 | - | KFJE, KFJF, KFG, KFK, KFM | Ever |
| Obstructive sleep apnea | DNPR | - | - | G473 | - | - | Ever |
| Family income | Statistics Denmark | - | - | - | - | - | - |
| Highest completed education | Statistics Denmark | - | - | - | - | - | - |
| Heart transplantation | DNPR | - | - | - | - | KFQA | Ever |

Abbreviations: DHFR = The Danish Heart Failure Registry; DNPR = The Danish National Patient Registry; The Danish National Prescription Registry = DPR; LVEF = ventricular ejection fraction; NYHA = New York Heart Association; ACE = angiotensin-converting enzyme; MRA = and mineralocorticoid receptor antagonists

*ICD code or ATC code

**Among patients without hypertension or with missing information on hypertension in in the DHFR, we updated the variable using DNPR and DPR, including an ICD code or at least 2 classes of medication.

#

# Baseline characteristics of cohorts

## Supplemental Table 3. Baseline characteristics of patients with no history of AF

| **Characteristics** | | | **N=27,947** |
| --- | --- | --- | --- |
| ***Demographics*** | | |  |
|  | Female sex, N (%) | | 9,397 (33.6) |
|  | Age, years, mean (SD) | | 68.5 (13.2) |
| ***Lifestyle factors*** | | |  |
|  | Elevated alcohol consumption, N (%) | | 2,187 (9.1) |
|  | Smoking status, N (%) | |  |
|  |  | Never | 6,516 (25.6) |
|  |  | Former | 10,983 (43.2) |
|  |  | Current | 7,941 (31.2) |
| ***Clinical characteristics*** | | |  |
|  | LVEF, N (%) | |  |
|  |  | <25% | 6,559 (24.0) |
|  |  | 25-40% | 17,047 (62.4) |
|  |  | >40-49% | 1,841 (6.7) |
|  |  | ≥50% | 1,883 (6.9) |
|  | NYHA class, N (%) | |  |
|  |  | I | 4,061 (16.1) |
|  |  | II | 15,264 (60.4) |
|  |  | III/IV | 5,964 (23.6) |
| ***Comorbidities and conditions*** | | |  |
|  | Myocardial infarction, N (%) | | 10,675 (38.2) |
|  | Any stroke, N (%) | | 2,698 (9.7) |
|  | Atrial fibrillation, N (%) | | 0 |
|  | Diabetes mellitus, N (%) | | 5,742 (20.6) |
|  | Chronic obstructive pulmonary disease, N (%) | | 3,568 (12.8) |
|  | Hypertension, N (%) | | 11,717 (41.9) |
|  | Chronic kidney disease, N (%) | | 2,082 (7.5) |
|  | Valvular disease, N (%) | | 2,567 (9.2) |
|  | Obesity, N (%) | | 2,270 (8.1) |
| ***Socioeconomic factors*** | | |  |
|  | Family income*, N (%) | |  |
|  |  | Lower (<49,626 euros) | 10,154 (36.6) |
|  |  | Medium (49,626-68,344 euros) | 9,036 (32.6) |
|  |  | Higher (>68,360 euros) | 8,549 (30.8) |
|  | Highest completed education, N (%) | |  |
|  |  | Lower | 11,697 (43.7) |
|  |  | Medium | 10.996 (41.0) |
|  |  | Higher | 4.099 (15.3) |

Missings values (%): alcohol: 3,864 (13.8), smoking: 2,507 (9.0), LVEF: 617 (2.2), NYHA: 2,658 (9.5), income: 208 (0.7), education 1.155 (4.3)

## Supplemental Table 4. Baseline characteristics of patients with no history of AF, by income

| **Characteristics** | | | **Income** | | | |
| --- | --- | --- | --- | --- | --- | --- |
|  |  |  | **Lower**  **N=10,154** | **Medium**  **N=9,036** | **Higher**  **N=8,549** | **Missing**  **N=208** |
| ***Demographics*** | | |  |  |  |  |
|  | Female sex, N (%) | | 3,874 (38.2) | 3,146 (34.8) | 2,300 (26,9) | 77 (27.0) |
|  | Age, years, mean (SD) | | 71.4 (13.7) | 69.3 (12.7) | 64.1 (11.8) | 77.9 (11.0) |
| ***Lifestyle factors*** | | |  |  |  |  |
|  | Elevated alcohol consumption, N (%) | | 744 (8.7) | 676 (8.6) | 753 (10.1) | 14 (9.1) |
|  | Smoking status, N (%) | |  |  |  |  |
|  |  | Never | 2,072 (22.7) | 2,041 (24.6) | 2,360 (30.1) | 43 (25.9) |
|  |  | Former | 3,898 (42.7) | 3,590 (43.3) | 3,435 (43.9) | 60 (36.1) |
|  |  | Current | 3,170 (34.7) | 2,670 (32.2) | 2,038 (26.0) | 63 (38.0) |
| ***Clinical characteristics*** | | |  |  |  |  |
|  | LVEF, N (%) | |  |  |  |  |
|  |  | <25% | 2,379 (24.2) | 2,168 (24.4) | 1,966 (23.3) | 36 (29.5) |
|  |  | 25-40% | 6,044 (61.5) | 5,554 (62.4) | 5,370 (63.5) | 79 (50.6) |
|  |  | >40-49% | 693 (7.1) | 578 (6.5) | 556 (6.6) | 14 (9.0) |
|  |  | ≥50% | 710 (7.2) | 595 (6.7) | 561 (6.6) | 17 (10.9) |
|  | NYHA class, N (%) | |  |  |  |  |
|  |  | I | 1,074 (12.1) | 1,242 (15.0) | 1,741 (21.7) | <5* |
|  |  | II | 5,180 (58.3) | 5,067 (61.3) | 4,986 (62.0) | 31 (34.8) |
|  |  | III/IV | 2,637 (29.7) | 1,961 (23.7) | 1,312 (16.3) | 55* (60.7) |
| ***Comorbidities and conditions*** | | |  |  |  |  |
|  | Myocardial infarction, N (%) | | 3,933 (38.7) | 3,474 (38.5) | 3,198 (37.4) | 70 (33.7) |
|  | Any stroke, N (%) | | 1,100 (10.8) | 964 (10.7) | 603 (7.1) | 31 (14.9) |
|  | Atrial fibrillation, N (%) | | 0 | 0 | 0 | 0 |
|  | Diabetes mellitus, N (%) | | 2,391 (23.6) | 1,966 (21.8) | 1,334 (15.6) | 51 (23.5) |
|  | Chronic obstructive pulmonary disease, N (%) | | 1,603 (15.8) | 1,265 (14.0) | 654 (7.7) | 45 (21.6) |
|  | Hypertension, N (%) | | 4,338 (42.7) | 4,021 (44.5) | 3,291 (38.5) | 67 (32.2) |
|  | Chronic kidney disease, N (%) | | 889 (8.8) | 704 (7.8) | 467 (5.5) | 22 (10.6) |
|  | Valvular disease, N (%) | | 993 (9.8) | 834 (9.2) | 703 (8.2) | 37 (17.8) |
|  | Obesity, N (%) | | 840 (8.3) | 815 (9.0) | 610 (7.1) | 5 (2.4) |
| ***Highest completed education, N (%)*** | | |  |  |  |  |
|  | Lower | | 5,613 (59.1) | 4,058 (46.4) | 1,938 (23.1) | 88 (55.7) |
|  | Medium | | 3,313 (34.9) | 3,783 (43.3) | 3,853 (45.9) | 47 (29.8) |
|  | Higher | | 569 (6.0) | 898 (10.3) | 2,609 (31.1) | 23 (14.6) |

*Numbers stated with a precision ±2 to adhere to applicable rules for discretion in the handling of data on an individual level.

Missings values (%): alcohol: 3,864 (13.8), smoking: 2,507 (9.0), LVEF: 617 (2.2), NYHA: 2,658 (9.5), education 1.155 (4.3)

## Supplemental Table 5. Baseline characteristics of patients with no history of AF, by education

| **Characteristics** | | | **Education** | | | |
| --- | --- | --- | --- | --- | --- | --- |
|  |  |  | **Lower**  **N=11,697** | **Medium**  **N=10,996** | **Higher**  **N=4,099** | **Missing**  **N=1,155** |
| ***Demographics*** | | |  |  |  |  |
|  | Female sex, N (%) | | 4,744 (40.6) | 2,836 (25.8) | 1,291 (31.5) | 526 (45.5) |
|  | Age, years, mean (SD) | | 70.1 (13.0) | 66.3 (12.6) | 67.1 (12.4) | 78.6 (16.1) |
| ***Lifestyle factors*** | | |  |  |  |  |
|  | Elevated alcohol consumption, N (%) | | 725 (7.2) | 1,031 (10.7) | 349 (9.9) | 82 (9.0) |
|  | Smoking status, N (%) | |  |  |  |  |
|  |  | Never | 2,504 (23.5) | 2,545 (25.1) | 1,158 (31.4) | 309 (33.2) |
|  |  | Former | 4,528 (42.4) | 4,421 (43.6) | 1,689 (45.8) | 345 (37.1) |
|  |  | Current | 3,644 (34.1) | 3,177 831.3) | 844 (22.9) | 276 (29.7) |
| ***Clinical characteristics*** | | |  |  |  |  |
|  | LVEF, N (%) | |  |  |  |  |
|  |  | <25% | 2,716 (23.8) | 2,636 (24.3) | 965 (23.9) | 242 (23.4) |
|  |  | 25-40% | 7,119 (62.3) | 6,757 (62.4) | 2,550 (63.2) | 621 (60.1) |
|  |  | >40-49% | 776 (7.8) | 726 (6.7) | 255 (6.3) | 84 (8.1) |
|  |  | ≥50% | 816 (7.1) | 718 (6.6) | 263 (6.5) | 86 (8.3) |
|  | NYHA class, N (%) | |  |  |  |  |
|  |  | I | 1,377 (13.1) | 1,887 (18.6) | 703 (18.5) | 83 (11.1) |
|  |  | II | 6,225 (59.4) | 6,211 (61.1) | 2,366 (62.3) | 462 (54.7) |
|  |  | III/IV | 2,876 (24.5) | 2,069 (20.4) | 730 (19.2) | 289 (34.2) |
| ***Comorbidities and conditions*** | | |  |  |  |  |
|  | Myocardial infarction, N (%) | | 4,594 (39.3) | 4,238 (38.5) | 1,445 (35.3) | 398 (34.6) |
|  | Any stroke, N (%) | | 1,241 (10.6) | 995 (9.1) | 344 (8.4) | 118 (10.2) |
|  | Atrial fibrillation, N (%) | | 0 | 0 | 0 | 0 |
|  | Diabetes mellitus, N (%) | | 2,645 (22.6) | 2,196 (20.0) | 670 (13.4) | 231 (20.0) |
|  | Chronic obstructive pulmonary disease, N (%) | | 1,847 (15.8) | 1,231 (11.2) | 361 (8.8) | 128 (11.1) |
|  | Hypertension, N (%) | | 4,997 (42.7) | 4,613 (42.0) | 1,591 (38.8) | 516 (44.7) |
|  | Chronic kidney disease, N (%) | | 995 (8.5) | 750 (6.8) | 255 (6.2) | 82 (7.1) |
|  | Valvular disease, N (%) | | 1,093 (9.3) | 947 (8.6) | 389 (9.5) | 138 (12.0) |
|  | Obesity, N (%) | | 1,082 (9.3) | 865 (8.9) | 251 (6.1) | 72 (6.2) |
| ***Family income, N (%)*** | | |  |  |  |  |
|  | Lower (<49,626 euros) | | 5,613 (48.4) | 3,313 (30.3) | 569 (14.0) | 659 (59.6) |
|  | Medium (49,626-68,344 euros) | | 4,058 (35.0) | 3784 (34.6) | 898 (22.0) | 297 (26.9) |
|  | Higher (>68,360 euros) | | 1.938 (16.7) | 3,853 (35.2) | 2,609 (64.0) | 149 (13.5) |

*Numbers stated with a precision ±2 to adhere to applicable rules for discretion in the handling of data on an individual level.

Missings values (%): alcohol: 3,864 (13.8), smoking: 2,507 (9.0), LVEF: 617 (2.2), NYHA: 2,658 (9.5), income: 208 (0.7)

##

## Supplemental Table 6. Baseline characteristics of patients with no history of myocardial infarction.

| **Characteristics** | | | **N=27,309** |
| --- | --- | --- | --- |
| ***Demographics*** | | |  |
|  | Female sex, N (%) | | 9,537 (34.9) |
|  | Age, years, mean (SD) | | 60.8 (13.1) |
| ***Lifestyle factors*** | | |  |
|  | Elevated alcohol consumption, N (%) | | 2,534 (10.8) |
|  | Smoking status, N (%) | |  |
|  |  | Never | 7,310 (29.9) |
|  |  | Former | 10,380 (42.4) |
|  |  | Current | 6,802 (27.8) |
| ***Clinical characteristics*** | | |  |
|  | LVEF, N (%) | |  |
|  |  | <25% | 7,516 (28.4) |
|  |  | 25-40% | 15,496 (58.4) |
|  |  | >40-49% | 1,644 (6.2) |
|  |  | ≥50% | 1,859 (7.0) |
|  | NYHA class, N (%) | |  |
|  |  | I | 3,408 (14.0) |
|  |  | II | 14,560 (59.8) |
|  |  | III/IV | 6,385 (26.2) |
| ***Comorbidities and conditions*** | | |  |
|  | Myocardial infarction, N (%) | | 0 |
|  | Any stroke, N (%) | | 2,896 (10.6) |
|  | Atrial fibrillation, N (%) | | 10,037 (36.8) |
|  | Diabetes mellitus, N (%) | | 5,193 (19.0) |
|  | Chronic obstructive pulmonary disease, N (%) | | 3,665 (13.4) |
|  | Hypertension, N (%) | | 11,682 (42.8) |
|  | Chronic kidney disease, N (%) | | 2,001 (7.3) |
|  | Valvular disease, N (%) | | 3,299 (12.1) |
|  | Obesity, N (%) | | 2,322 (8.5) |
| ***Socioeconomic factors*** | | |  |
|  | Family income, N (%) | |  |
|  |  | Lower (<49,883 euros) | 9,987 (36.9) |
|  |  | Medium (49,883-68,916 euros) | 8,785 (32.5) |
|  |  | Higher (>68,918 euros) | 8,289 (30.6) |
|  | Highest completed education, N (%) | |  |
|  |  | Lower | 11,225 (43.0) |
|  |  | Medium | 10,463 (40.1) |
|  |  | Higher | 4,397 (16.9) |

Missings values (%): alcohol: 3,798 (13.9), smoking: 2,817 (10.3), LVEF: 794 (2.9), NYHA: 2,956 (10.8), income: 248 (0.9), education 1.224 (4.5)

## Supplemental Table 7. Baseline characteristics of patients with no history of myocardial infarction, by income

| **Characteristics** | | | **Income** | | | |
| --- | --- | --- | --- | --- | --- | --- |
|  |  |  | **Lower**  **N=9,987** | **Medium**  **N=8,785** | **Higher**  **N=8,298** | **Missing**  **N=248** |
| ***Demographics*** | | |  |  |  |  |
|  | Female sex, N (%) | | 3,974 (38.2) | 3,146 (34.8) | 2,300 (26.9) | 77 (37.0) |
|  | Age, years, mean (SD) | | 72.6 (13.3) | 70.2 (12.7) | 65.7 (12.1) | 78.6 (11.3) |
| ***Lifestyle factors*** | | |  |  |  |  |
|  | Elevated alcohol consumption, N (%) | | 846 (10.0) | 783 (10.3) | 891 (12.3) | 14 (7.6) |
|  | Smoking status, N (%) | |  |  |  |  |
|  |  | Never | 2,378 (27.0) | 2,287 (28.8) | 2,585 (34.4) | 60 (31.6) |
|  |  | Former | 3,672 (41.6) | 3,365 (42.3) | 3,269 (43.5) | 74 (39.0) |
|  |  | Current | 2,774 (31.4) | 2,303 (29.0) | 1,669 (22.2) | 56 (29.5) |
| ***Clinical characteristics*** | | |  |  |  |  |
|  | LVEF, N (%) | |  |  |  |  |
|  |  | <25% | 2,656 (27.8) | 2,452 (28.5) | 2,363 (28.9) | 45 (26.2) |
|  |  | 25-40% | 5,460 (57.2) | 5,086 (59.0) | 4,868 (59.5) | 82 (47.7) |
|  |  | >40-49% | 688 (7.2) | 503 (5.8) | 430 (5.3) | 23 (13.4) |
|  |  | ≥50% | 737 (7.7) | 577 (6.7) | 523 (6.4) | 22 (12.8) |
|  | NYHA class, N (%) | |  |  |  |  |
|  |  | I | 873 (10.2) | 1,075 (13.5) | 1,455 (18.8) | 5 (4.6) |
|  |  | II | 4,927 (57.8) | 4,803 (60.2) | 4,789 (61.9) | 31 (37.6) |
|  |  | III/IV | 2,723 (32.0) | 2,100 (26.3) | 1,499 (19.4) | 63 (57.8) |
| ***Comorbidities and conditions*** | | |  |  |  |  |
|  | Myocardial infarction, N (%) | | 0 | 0 | 0 | 0 |
|  | Any stroke, N (%) | | 1,122 (11.2) | 1,022 (11.6) | 710 (8.6) | 42 (16.9) |
|  | Atrial fibrillation, N (%) | | 3,632 (36.5) | 3,240 (26.9) | 3,045 (36.7) | 110 (44.4) |
|  | Diabetes mellitus, N (%) | | 2,151 (21.5) | 1,840 (20.9) | 1,156 (14.0) | 46 (18.6) |
|  | Chronic obstructive pulmonary disease, N (%) | | 1,601 (16.0) | 1,312 (14.9) | 705 (8.5) | 47 (19.0) |
|  | Hypertension, N (%) | | 4,341 (43.5) | 3,968 (45.2) | 3,286 (39.6) | 87 (35.1) |
|  | Chronic kidney disease, N (%) | | 834 (8.4) | 678 (7.7) | 467 (5.6) | 22 (8.9) |
|  | Valvular disease, N (%) | | 1,252 (12.5) | 1,026 (11.7) | 970 (11.7) | 51 (20.6) |
|  | Obesity, N (%) | | 871 (8.7) | 859 (9.8) | 583 (7.0) | 9 (3.6) |
| ***Highest completed education, N (%)*** | | |  |  |  |  |
|  | Lower | | 5,501 (59.3) | 3,886 (45.8) | 1,730 (21.3) | 108 (59.3) |
|  | Medium | | 3,204 (34.5) | 3,633 (42.8) | 3,577 (44.0) | 49 (26.9) |
|  | Higher | | 577 (6.2) | 974 (11.5) | 2,821 (34.7) | 25 (13.7) |

Missings values (%): alcohol: 3,798 (13.9), smoking: 2,817 (10.3), LVEF: 794 (2.9), NYHA: 2,956 (10.8), education 1.224 (4.5)

##

## Supplemental Table 8. Baseline characteristics of patients with no history of myocardial infarction, by education

| **Characteristics** | | | **Education** | | | |
| --- | --- | --- | --- | --- | --- | --- |
|  |  |  | **Lower**  **N=11,225** | **Medium**  **N=10,463** | **Higher**  **N=4,397** | **Missing**  **N=1,224** |
| ***Demographics*** | | |  |  |  |  |
|  | Female sex, N (%) | | 4,745 (42.3) | 2,790 (26.7) | 1,377 (31.3) | 625 (51.1) |
|  | Age, years, mean (SD) | | 71.4 (12.8) | 67.4 (12.5) | 68.5 (12.2) | 80.8 (15.2) |
| ***Lifestyle factors*** | | |  |  |  |  |
|  | Elevated alcohol consumption, N (%) | | 786 (8.2) | 1,153 (12.6) | 514 (13.5) | 81 (8.4) |
|  | Smoking status, N (%) | |  |  |  |  |
|  |  | Never | 2,797 (27.8) | 2,736 (28.7) | 1,391 (35.3) | 386 (39.8) |
|  |  | Former | 4,189 (41.7) | 4,079 (42.8) | 1,753 (44.5) | 359 (37.1) |
|  |  | Current | 3,066 (30.5) | 2,720 (28.5) | 792 (20.1) | 224 (23.1) |
| ***Clinical characteristics*** | | |  |  |  |  |
|  | LVEF, N (%) | |  |  |  |  |
|  |  | <25% | 3,046 (28.0) | 2,977 (29.0) | 1,221 (28.3) | 272 (26.2) |
|  |  | 25-40% | 6,358 (58.4) | 5,996 (58.4) | 2,583 (59.8) | 559 (53.8) |
|  |  | >40-49% | 675 (6.2) | 630 (6.1) | 241 (5.6) | 98 (9.4) |
|  |  | ≥50% | 808 (7.4) | 668 (6.5) | 272 (6.3) | 111 (10.7) |
|  | NYHA class, N (%) | |  |  |  |  |
|  |  | I | 1,133 (11.4) | 1,507 (15.8) | 690 (17.1) | 78 (9.3) |
|  |  | II | 5,748 (58.0) | 5,871 (61.4) | 2,483 (61.6) | 458 (54.3) |
|  |  | III/IV | 3,035 (30.6) | 2,184 (22.8) | 859 (21.3) | 307 (36.4) |
| ***Comorbidities and conditions*** | | |  |  |  |  |
|  | Myocardial infarction, N (%) | | 0 | 0 | 0 | 0 |
|  | Any stroke, N (%) | | 1,292 (11.5) | 1,017 (9.7) | 454 (10.3) | 133 (10.9) |
|  | Atrial fibrillation, N (%) | | 4,122 (36.7) | 3,705 (35.4) | 1,743 (39.6) | 467 (38.2) |
|  | Diabetes mellitus, N (%) | | 2,353 (21.0) | 1,969 (18.8) | 651 (14.8) | 220 (18.0) |
|  | Chronic obstructive pulmonary disease, N (%) | | 1,840 (16.4) | 1,263 (12.1) | 415 (9.4) | 147 (12.0) |
|  | Hypertension, N (%) | | 4,892 (43.6) | 4,461 (42.6) | 1,782 (40.5) | 547 (44.7) |
|  | Chronic kidney disease, N (%) | | 915 (8.2) | 721 (6.9) | 263 (6.0) | 102 (8.3) |
|  | Valvular disease, N (%) | | 1,350 (12.0) | 1,215 (11.6) | 557 (12.7) | 177 (14.5) |
|  | Obesity, N (%) | | 1,096 (9.8) | 864 (8.3) | 291 (6.6) | 71 (5.8) |
| ***Family income, N (%)*** | | |  |  |  |  |
|  | Lower (<49,883 euros) | | 5,501 (49.5) | 3,204 (30.8) | 577 (13.2) | 705 (60.9) |
|  | Medium (49,883-68,916 euros) | | 3,886 (35.0) | 3,633 (34.9) | 974 (22.3) | 292 (25.2) |
|  | Higher (>68,918 euros) | | 1,730 (15.6) | 3,577 (34.4) | 2,821 (64.5) | 161 (13.9) |

*Numbers stated with a precision ±2 to adhere to applicable rules for discretion in the handling of data on an individual level.

Missings values (%): alcohol: 3,864 (13.8), smoking: 2,507 (9.0), LVEF: 617 (2.2), NYHA: 2,658 (9.5), income: 208 (0.7)

##

## Supplemental Table 9. Baseline characteristics of patients with no history of any stroke.

| **Characteristics** | | | **N=36,801** |
| --- | --- | --- | --- |
| ***Demographics*** | | |  |
|  | Female sex, N (%) | | 12,068 (32.8) |
|  | Age, years, mean (SD) | | 69.8 (12.9) |
| ***Lifestyle factors*** | | |  |
|  | Elevated alcohol consumption, N (%) | | 2,984 (9.4) |
|  | Smoking status, N (%) | |  |
|  |  | Never | 9,213 (27.7) |
|  |  | Former | 14,731 (44.2) |
|  |  | Current | 9,376 (28.1) |
| ***Clinical characteristics*** | | |  |
|  | LVEF, N (%) | |  |
|  |  | <25% | 8,690 (24.2) |
|  |  | 25-40% | 22,232 (61.9) |
|  |  | >40-49% | 2,392 (6.7) |
|  |  | ≥50% | 2,578 (8.2) |
|  | NYHA class, N (%) | |  |
|  |  | I | 5,051 (15.2) |
|  |  | II | 19,988 (60.1) |
|  |  | III/IV | 8,237 (25.8) |
| ***Comorbidities and conditions*** | | |  |
|  | Myocardial infarction, N (%) | | 12,388 (33.7) |
|  | Any stroke, N (%) | | 0 |
|  | Atrial fibrillation, N (%) | | 11,552 (31.4) |
|  | Diabetes mellitus, N (%) | | 7,295 (19.8) |
|  | Chronic obstructive pulmonary disease, N (%) | | 4,814 (13.1) |
|  | Hypertension, N (%) | | 15,409 (41.9) |
|  | Chronic kidney disease, N (%) | | 2,787 (7.6) |
|  | Valvular disease, N (%) | | 4,133 (11.2) |
|  | Obesity, N (%) | | 3,164 (8.6) |
| ***Socioeconomic factors*** | | |  |
|  | Family income, N (%) | |  |
|  |  | Lower (<49,726 euros) | 9,987 (36.9) |
|  |  | Medium (49,726-68,664 euros) | 8,785 (32.5) |
|  |  | Higher (>68,667 euros) | 8,289 (30.6) |
|  | Highest completed education, N (%) | |  |
|  |  | Lower | 15,287 (43.4) |
|  |  | Medium | 14,283 (40.6) |
|  |  | Higher | 5,646 (16.0) |

Missing values (%): alcohol 4,970 (13.5), smoking: 3,481 (9.5), LVEF: 909 (2.5), NYHA: 3,525 (9.6), income: 298 (0.8), education 1,585 (4.3)

## Supplemental Table 10. Baseline characteristics of patients with no history of any stroke, by income.

| **Characteristics** | | | **Income** | | | |
| --- | --- | --- | --- | --- | --- | --- |
|  |  |  | **Lower**  **N=13,454** | **Medium**  **N=11,856** | **Higher**  **N=11,193** | **Missing**  **N=298** |
| ***Demographics*** | | |  |  |  |  |
|  | Female sex, N (%) | | 5,153 (38.3) | 3,986 (33.6) | 2,804 (25.1) | 125 (42.0) |
|  | Age, years, mean (SD) | | 72.7 (13.0) | 70.4 (12.5) | 65.3 (11.8) | 79.2 (11.1) |
| ***Lifestyle factors*** | | |  |  |  |  |
|  | Elevated alcohol consumption, N (%) | | 969 (8.5) | 918 (8.9) | 1,081 (11.0) | 16 (7.4) |
|  | Smoking status, N (%) | |  |  |  |  |
|  |  | Never | 3,031 (25.2) | 2,849 (26.3) | 3,257 (31.8) | 76 (33.2) |
|  |  | Former | 5,251 (43.7) | 4,844 (44.7) | 4,551 (44.4) | 85 (37.1) |
|  |  | Current | 3,727 (31.0) | 3,149 (29.0) | 2,432 (23.8) | 68 (29.7) |
| ***Clinical characteristics*** | | |  |  |  |  |
|  | LVEF, N (%) | |  |  |  |  |
|  |  | <25% | 3,102 (23.9) | 2,858 (24.5) | 2,668 (24.1) | 62 (28.4) |
|  |  | 25-40% | 7,901 (61.0) | 7,247 (62.2) | 6,979 (63.1) | 105 (48.2) |
|  |  | >40-49% | 939 (7.3) | 741 (6.4) | 685 (6.2) | 27 (13.4) |
|  |  | ≥50% | 1,016 (7.8) | 801 (6.9) | 737 (6.7) | 24 (11.0) |
|  | NYHA class, N (%) | |  |  |  |  |
|  |  | I | 1,292 (11.0) | 1,559 (14.3) | 2,194 (20.8) | 6 (4.5) |
|  |  | II | 6,787 (57.9) | 6,648 (61.1) | 6,508 (61.8) | 45 (33.8) |
|  |  | III/IV | 3,641 (31.1) | 2,678 (24.6) | 1,836 (17.4) | 82 (61.7) |
| ***Comorbidities and conditions*** | | |  |  |  |  |
|  | Myocardial infarction, N (%) | | 4,706 (35.0) | 4,038 (34.1) | 3,552 (31.7) | 92 (30.9) |
|  | Any stroke, N (%) | | 0 | 0 | 0 | 0 |
|  | Atrial fibrillation, N (%) | | 4,333 (32.2) | 3,768 (31.8) | 3,330 (29.8) | 121 (40.6) |
|  | Diabetes mellitus, N (%) | | 3,094 (23.0) | 2,509 (21.2) | 1,631 (14.6) | 61 (20.5) |
|  | Chronic obstructive pulmonary disease, N (%) | | 2,150 (16.0) | 1,731 (14.6) | 871 (7.8) | 62 (20.8) |
|  | Hypertension, N (%) | | 5,762 (42.8) | 5,246 (44.3) | 4,303 (38.4) | 98 (32.9) |
|  | Chronic kidney disease, N (%) | | 1,197 (8.9) | 936 (7.9) | 626 (5.6) | 28 (9.4) |
|  | Valvular disease, N (%) | | 1,628 (12.1) | 1,319 (11.1) | 1,128 (10.1) | 58 (19.5) |
|  | Obesity, N (%) | | 1,206 (9.0) | 1,146 (9.7) | 802 (7.2) | 10 (3.4) |
| ***Highest completed education, N (%)*** | | |  |  |  |  |
|  | Lower | | 7,496 (59.7) | 5,238 (45.8) | 2,429 (22.1) | 124 (57.7) |
|  | Medium | | 4,327 (34.5) | 4,938 (43.2) | 4,957 (45.1) | 61 (28.4) |
|  | Higher | | 739 (5.9) | 1,264 (11.1) | 3,613 (32.9) | 30 (14.0) |

Missings values (%): alcohol 4,970 (13.5), smoking: 3,481 (9.5), LVEF: 909 (2.5), NYHA: 3,525 (9.6), education 1,585 (4.3)

## Supplemental Table 11. Baseline characteristics of patients with no history of any stroke, by education

| **Characteristics** | | | **Education** | | | |
| --- | --- | --- | --- | --- | --- | --- |
|  |  |  | **Lower**  **N=15,287** | **Medium**  **N=14,283** | **Higher**  **N=5,646** | **Missing**  **N=1,585** |
| ***Demographics*** | | |  |  |  |  |
|  | Female sex, N (%) | | 6,173 (40.4) | 3,517 (24.6) | 1,637 (29.0) | 741 (46.8) |
|  | Age, years, mean (SD) | | 71.4 (12.6) | 67.4 (12.3) | 68.4 (12.2) | 80.4 (15.3) |
| ***Lifestyle factors*** | | |  |  |  |  |
|  | Elevated alcohol consumption, N (%) | | 936 (7.1) | 1,384 (11.0) | 564 (11.6) | 100 (8.1) |
|  | Smoking status, N (%) | |  |  |  |  |
|  |  | Never | 3,580 (25.8) | 3,490 (26.6) | 1,674 (33.0) | 469 (37.1) |
|  |  | Former | 6,025 (43.5) | 5,871 (44.7) | 2,340 (36.3) | 486 (38.4) |
|  |  | Current | 4,250 (30.7) | 3,762 (28.7) | 1,054 (20.8) | 310 (24.5) |
| ***Clinical characteristics*** | | |  |  |  |  |
|  | LVEF, N (%) | |  |  |  |  |
|  |  | <25% | 3,544 (23.8) | 3,424 (24.4) | 1,394 (25.1) | 328 (23.8) |
|  |  | 25-40% | 9,207 (61.8) | 8,751 (62.2) | 3,477 (62.6) | 979 (57.8) |
|  |  | >40-49% | 997 (6.7) | 945 (6.7) | 330 (5.9) | 120 (8.7) |
|  |  | ≥50% | 1,149 (7.7) | 943 (6.7) | 352 (6.3) | 134 (9.7) |
|  | NYHA class, N (%) | |  |  |  |  |
|  |  | I | 1,678 (12.2) | 2,294 (17.4) | 961 (18.4) | 118 (10.4) |
|  |  | II | 8,075 (58.9) | 8,084 (61.2) | 3215 (61.5) | 614 (54.1) |
|  |  | III/IV | 3,952 (28.8) | 2,831 (21.4) | 1,052 (20.1) | 402 (35.5) |
| ***Comorbidities and conditions*** | | |  |  |  |  |
|  | Myocardial infarction, N (%) | | 5,354 (35.0) | 4,837 (33.9) | 1,703 (30.2) | 949 (31.2) |
|  | Any stroke, N (%) | | 0 | 0 | 0 | 0 |
|  | Atrial fibrillation, N (%) | | 4,831 (31.6) | 4,282 (30.0) | 1,891 (33.5) | 548 (34.6) |
|  | Diabetes mellitus, N (%) | | 3,363 (22.0) | 2,782 (19.5) | 865 (15.3) | 285 (18.0) |
|  | Chronic obstructive pulmonary disease, N (%) | | 2,475 (16.2) | 1,656 (11.6) | 498 (8.8) | 185 (11.7) |
|  | Hypertension, N (%) | | 6,536 (42.8) | 5,956 (41.7) | 2,215 (39.2) | 702 (44.3) |
|  | Chronic kidney disease, N (%) | | 1,289 (8.4) | 1,021 (7.2) | 352 (6.2) | 125 (7.9) |
|  | Valvular disease, N (%) | | 1m759 (11.5) | 1,531 (10.7) | 632 (11.2) | 211 (13.3) |
|  | Obesity, N (%) | | 1,486 (9.7) | 1,228 (8.6) | 352 (6.2) | 98 (6.2) |
| ***Family income, N (%)*** | | |  |  |  |  |
|  | Lower (<49,726 euros) | | 7,496 (49.4) | 3,427 (30.4) | 839 (13.2) | 892 (59.4) |
|  | Medium (49,726-68,664 euros) | | 5,238 (34.5) | 4,938 (34.7) | 1,274 (22.5) | 416 (27.7) |
|  | Higher (>68,667 euros) | | 2,429 (16.0) | 4,957 (34.9) | 3,613 (64.3) | 193 (12.9) |

Missings values (%): alcohol 4,970 (13.5), smoking: 3,481 (9.5), LVEF: 909 (2.5), NYHA: 3,525 (9.6), education 1,585 (4.3)

## Supplemental table 12. Baseline characteristics of all patients, by income.

| **Characteristics** | | | **Income** | | | |
| --- | --- | --- | --- | --- | --- | --- |
|  |  |  | **Lower**  **N=15,173** | **Medium**  **N=13,317** | **Higher**  **N=12,542** | **Missing**  **N=366** |
| ***Demographics*** | | |  |  |  |  |
|  | Female sex, N (%) | | 5,727 (37.7) | 4,456 (33.5) | 3,122 (24.9) | 151 (41.3) |
|  | Age, years, mean (SD) | | 73.0 (12.8) | 70.8 (12.3) | 65.8 (11.9) | 79.0 (10.8) |
| ***Lifestyle factors*** | | |  |  |  |  |
|  | Elevated alcohol consumption, N (%) | | 1,100 (8.6) | 1,029 (8.9) | 1,186 (10.8) | 21 (8.0) |
|  | Smoking status, N (%) | |  |  |  |  |
|  |  | Never | 3,387 (25.0) | 3,142 (25.9) | 3,589 (31.4) | 88 (31.1) |
|  |  | Former | 5,960 (44.0) | 5,422 (44.7) | 5,146 (45.0) | 113 (39.9) |
|  |  | Current | 4,188 (30.9) | 3,568 (29.4) | 2,713 (23.7) | 82 (29.0) |
| ***Clinical characteristics*** | | |  |  |  |  |
|  | LVEF, N (%) | |  |  |  |  |
|  |  | <25% | 3,485 (23.9) | 3,190 (23.4) | 2,996 (24.2) | 79 (29.0) |
|  |  | 25-40% | 8,888 (60.9) | 8,147 (62.4) | 7,815 (63.1) | 135 (49.6) |
|  |  | >40-49% | 1,078 (8.4) | 836 (6.4) | 755 (6.1) | 31 (11.4) |
|  |  | ≥50% | 1,134 (7.8) | 982 (6.8) | 821 (6.6) | 28 (9.9) |
|  | NYHA class, N (%) | |  |  |  |  |
|  |  | I | 1,422 (10.8) | 1,683 (13.9) | 2,380 (20.2) | 7 (4.1) |
|  |  | II | 7,586 (57.8) | 7,379 (60.9) | 7,271 (61.8) | 59 (34.9) |
|  |  | III/IV | 4,125 (31.4) | 3,061 (25.3) | 2,120 (18.0) | 103 (61.0) |
| ***Comorbidities and conditions*** | | |  |  |  |  |
|  | Myocardial infarction, N (%) | | 5,391 (35.5) | 4,599 (34.5) | 3,981 (31.7) | 118 (32.2) |
|  | Any stroke, N (%) | | 1,836 (12.1) | 1,601 (12.0) | 1,092 (8.7) | 68 (18.6) |
|  | Atrial fibrillation, N (%) | | 5,038 (33.2) | 4,376 (32.9) | 3,879 (30.9) | 158 (43.2) |
|  | Diabetes mellitus, N (%) | | 3,626 (23.9) | 2,964 (22.3) | 1,920 (15.3) | 80 (21.9) |
|  | Chronic obstructive pulmonary disease, N (%) | | 2,446 (16.1) | 2,000 (15.0) | 1,021 (8.1) | 76 (20.8) |
|  | Hypertension, N (%) | | 6,659 (43.9) | 6,059 (45.5) | 4,930 (39.3) | 126 (34.4) |
|  | Chronic kidney disease, N (%) | | 1,422 (9.4) | 1,137 (8.5) | 757 (6.0) | 42 (11.5) |
|  | Valvular disease, N (%) | | 1,882 (12.4) | 1,521 (11.4) | 1,312 (10.5) | 72 (19.7) |
|  | Obesity, N (%) | | 1,380 (9.1) | 1,273 (6.6) | 906 (7.2) | 13 (3.6) |
| ***Highest completed education, N (%)*** | | |  |  |  |  |
|  | Lower | | 8,493 (59.9) | 5,933 (46.1) | 2,782 (22.6) | 157 (57.9) |
|  | Medium | | 4,845 (34.2) | 5,537 (43.1) | 5,481 (44.5) | 76 (28.0) |
|  | Higher | | 833 (5.9) | 1,389 (10.8) | 4,049 (32.9) | 38 (14.0) |

Missings values (%): alcohol: 5,641 (13.6), smoking: 4,000 (9.7), LVEF: 1,089 (2.6), NYHA: 4,202 (10.2), education 1,785 (4.3)

## Supplemental Table 13. Baseline characteristics of all patients, by education

| **Characteristics** | | | **Education** | | | |
| --- | --- | --- | --- | --- | --- | --- |
|  |  |  | **Lower**  **N=17,365** | **Medium**  **N=15,939** | **Higher**  **N=6,309** | **Missing**  **N=1,785** |
| ***Demographics*** | | |  |  |  |  |
|  | Female sex, N (%) | | 6,956 (40.1) | 3,863 (24.2) | 1,806 (28.6) | 831 (46.6) |
|  | Age, years, mean (SD) | | 71.7 (12.4) | 67.9 (12.2) | 68.8 (12.0) | 80.6 (15.1) |
| ***Lifestyle factors*** | | |  |  |  |  |
|  | Elevated alcohol consumption, N (%) | | 1,059 (7.1) | 1,538 (11.0) | 626 (11.5) | 113 (8.1) |
|  | Smoking status, N (%) | |  |  |  |  |
|  |  | Never | 4,026 (25.7) | 3,809 (26.1) | 1,848 (32.6) | 523 (36.9) |
|  |  | Former | 6,842 (43.6) | 6,602 (45.2) | 2,652 (46.7) | 545 (38.5) |
|  |  | Current | 4,826 (30.8) | 4,201 (28.8) | 1,175 (20.7) | 349 (24.6) |
| ***Clinical characteristics*** | | |  |  |  |  |
|  | LVEF, N (%) | |  |  |  |  |
|  |  | <25% | 4,011 (23.7) | 3,834 (24.5) | 1,533 (24.7) | 372 (23.0) |
|  |  | 25-40% | 10,452 (61.9) | 9,754 (62.3) | 3,894 (62.9) | 885 (9.2) |
|  |  | >40-49% | 1,149 (6.8) | 1,036 (6.6) | 372 (6.0) | 143 (9.2) |
|  |  | ≥50% | 1,288 (7.6) | 1,040 (6.6) | 397 (6.4) | 149 (9.6) |
|  | NYHA class, N (%) | |  |  |  |  |
|  |  | I | 1,863 (12.1) | 2,469 (16.8) | 1,026 (17.7) | 134 (10.6) |
|  |  | II | 9,041 (58.5) | 8,974 (61.2) | 3,603 (62.0) | 677 (53.8) |
|  |  | III/IV | 4,557 (29.5) | 3,221 (22.0) | 1,183 (20.4) | 448 (35.6) |
| ***Comorbidities and conditions*** | | |  |  |  |  |
|  | Myocardial infarction, N (%) | | 6,140 (35.4) | 5,476 (34.4) | 1,912 (30.3) | 561 (31.4) |
|  | Any stroke, N (%) | | 2,078 (12.0) | 1,656 (10.4) | 663 (10.5) | 200 (11.2) |
|  | Atrial fibrillation, N (%) | | 5,668 (32.6) | 4,943 (31.0) | 2,210 (35.0) | 630 (25.3) |
|  | Diabetes mellitus, N (%) | | 4,005 (23.1) | 3,233 (20.4) | 1,010 (16.0) | 342 (19.2) |
|  | Chronic obstructive pulmonary disease, N (%) | | 2,860 (16.5) | 1,900 (11.9) | 576 (9.1) | 207 (11.6) |
|  | Hypertension, N (%) | | 7,644 (44.0) | 6,795 (42.6) | 2,536 (40.2) | 799 (44.8) |
|  | Chronic kidney disease, N (%) | | 1,596 (9.0) | 1,218 (7.6) | 422 (6.7) | 149 (8.4) |
|  | Valvular disease, N (%) | | 2,051 (11.8) | 1,755 (11.0) | 743 (11.8) | 238 (13.3) |
|  | Obesity, N (%) | | 1,708 (9.8) | 1,350 (8.5) | 402 (6.4) | 112 (6.3) |
| ***Family income, N (%)*** | | |  |  |  |  |
|  | Lower (<49,606 euros) | | 8,493 (49.4) | 4,845 (30.5) | 833 (13.3) | 1,002 (59.3) |
|  | Medium (49,608-68,011 euros) | | 5,933 (34.5) | 5,537 (34.9) | 1,389 (22.2) | 458 (27.1) |
|  | Higher (>68,012 euros) | | 2,782 (16.2) | 5,481 (34.6) | 4,049 (64.6) | 230 (13.6) |

Missings values (%): alcohol: 5,641 (13.6), smoking: 4,000 (9.7), LVEF: 1,089 (2.6), NYHA: 4,202 (10.2), income: 366 (0.9)

# Atrial fibrillation

## Supplemental Table 14. Hazard ratios with 95% CI for incident AF and interaction between income and education in the multivariable-adjusted* models.

|  |  | **Income** |  |  |
| --- | --- | --- | --- | --- |
|  |  | **Lower** | **Medium** | **Higher** |
| **Education** | **Lower** | 0.90 (0.80-1.01) | 0.93 (0.82-1.06) | 0.99 (0.85-1.15) |
|  | **Medium** | 0.96 (0.85-1.09) | 0.91 (0.80-1.03) | 0.89 (0.78-1.01) |
|  | **Higher** | 1.08 (0.87-1.34) | 0.90 (0.74-1.09) | 1.00 (ref) |
| ***By level of income*** | |  |  |  |
| **Education** | **Lower** | 0.83 (0.68-1.02) | 1.05 (0.87-1.26) | 1.01 (0.87-1.18) |
|  | **Medium** | 0.91 (0.73-1.13) | 1.01 (0.84-1.22) | 0.91 (0.79-1.03) |
|  | **Higher** | 1.00 (ref) | 1.00 (ref) | 1.00 (ref) |

P-value for interaction: 0.203

* Adjusted for all covariates listed in Table 1 except AF

## Supplement Table 15. Hazard ratios with 95% CI for the association between socioeconomic factors and incident AF, by sex.

|  | **Women** | | | **Men** | | | **P value*** |
| --- | --- | --- | --- | --- | --- | --- | --- |
|  | **Age-adjusted** | **Model 1** | **Model 2** | **Age-adjusted** | **Model 1** | **Model 2** |  |
| **Income** |  |  |  |  |  |  | 0.005 |
| Lower | 1.13 (0.97-1.32) | 1.10 (0.95-1.29) | 1.11 (0.95-1.30) | 0.96 (0.88-1.05) | 0.92 (0.84-1.00) | 0.94 (0.86-1.02) |  |
| Medium | 0.99 (0.84-1.17) | 0.97 (0.82-1.14) | 0.97 (0.83-1.15) | 0.99 (0.91-1.08) | 0.96 (0.88-1.05) | 0.98 (0.89-1.07) |  |
| Higher | 1.00 (ref) | 1.00 (ref) | 1.00 (ref) | 1.00 (ref) | 1.00 (ref) | 1.00 (ref) |  |
| **Education** |  |  |  |  |  |  | 0.67 |
| Lower | 0.96 (0.81-1.14) | 0.94 (0.79-1.12) | 0.95 (0.80-1.14) | 0.92 (0.83-1.02) | 0.90 (0.81-1.00) | 0.92 (0.83-1.02) |  |
| Medium | 0.97 (0.80-1.17) | 0.97 (0.80-1.17) | 0.98 (0.81-1.18) | 0.91 (0.82-1.01) | 0.91 (0.82-1.01) | 0.92 (0.83-1.01) |  |
| Higher | 1.00 (ref) | 1.00 (ref) | 1.00 (ref) | 1.00 (ref) | 1.00 (ref) | 1.00 (ref) |  |

Model 1: Adjusted for all covariates listed in Table 1 except outcome of interest, smoking and alcohol

Model 2: Adjusted for all covariates listed in Table 1 except outcome of interest.

*P-values for interaction by sex in fully-adjusted model

# Myocardial infarction

## Supplemental Table 16. Hazard ratios with 95% CI for incident myocardial infarction and interaction between income and education in the multivariable-adjusted* models.

|  |  | **Income** |  |  |
| --- | --- | --- | --- | --- |
|  |  | **Lower** | **Medium** | **Higher** |
| **Education** | **Lower** | 1.38 (1.10-1.72) | 1.24 (0.97-1.57) | 1.17 (0.87-1.57) |
|  | **Medium** | 1.36 (1.07-1.72) | 1.17 (0.92-1.50) | 1.07 (0.83-1.38) |
|  | **Higher** | 1.33 (0.90-1.97) | 1.00 (0.69-1.44) | 1.00 (ref) |
| ***By level of income*** | | | | |
| **Education** | **Lower** | 1.04 (0.72-1.49) | 1.25 (0.89-1.77) | 1.15 (0.85-1.55) |
|  | **Medium** | 1.01 (0.70-1.47) | 1.20 (0.85-1.70) | 1.06 (0.81-1.37) |
|  | **Higher** | 1.00 (ref) | 1.00 (ref) | 1.00 (ref) |

P-value for interaction: 0.952

*Adjusted for all covariates listed in Table 1 except myocardial infarction

## Supplement Table 17. Hazard ratios with 95% CI for the association between socioeconomic factors and incident myocardial infarction, by sex.

|  | **Women** | | | **Men** | | | **P value*** |
| --- | --- | --- | --- | --- | --- | --- | --- |
|  | **Age-adjusted** | **Model 1** | **Model 2** | **Age-adjusted** | **Model 1** | **Model 2** |  |
| **Income** |  |  |  |  |  |  | 0.92 |
| Lower | 1.36 (1.05-1.75) | 1.28 (0.99-1.76) | 1.24 (0.95-1.61) | 1.49 (1.26-1.75) | 1.35 (1.14-1.59) | 1.31 (1.11-1.55) |  |
| Medium | 1.19 (0.91-1.57) | 1.15 (0.87-1.51) | 1.12 (0.85-1.47) | 1.21 (1.02-1.44) | 1.13 (0.95-1.34) | 1.11 (0.93-1.32) |  |
| Higher | 1.00 (ref) | 1.00 (ref) | 1.00 (ref) | 1.00 (ref) | 1.00 (ref) | 1.00 (ref) |  |
| **Education** |  |  |  |  |  |  | 0.55 |
| Lower | 1.18 (0.89-1.58) | 1.11 (0.83-1.49) | 1.06 (0.79-1.42) | 1.49 (1.21-1.82) | 1.35 (1.10-1.66) | 1.32 (1.07-.62) |  |
| Medium | 1.10 (0.81-1.49) | 1.05 (0.77-1.42) | 1.02 (0.75-1.39) | 1.31 (1.06-1.60) | 1.23 (1.00-1.51) | 1.21 (0.98-1.49) |  |
| Higher | 1.00 (ref) | 1.00 (ref) | 1.00 (ref) | 1.00 (ref) | 1.00 (ref) | 1.00 (ref) |  |

Model 1: Adjusted for all covariates listed in Table 1 except outcome of interest, smoking and alcohol

Model 2: Adjusted for all covariates listed in Table 1 except outcome of interest.

*P-values for interaction by sex in fully-adjusted model

# Any stroke

## Supplemental Table 18. Hazard ratios with 95% CI for incident stroke and interaction between income and education in the multivariable-adjusted* models.

|  |  | **Income** |  |  |
| --- | --- | --- | --- | --- |
|  |  | **Lower** | **Medium** | **Higher** |
| **Education** | **Lower** | 1.25 (1.04-1.49) | 1.15 (0.95-1.39) | 0.88 (0.68-1.14) |
|  | **Medium** | 1.30 (1.08-1.58) | 1.16 (0.96-1.41) | 0.89 (0.72-1.10) |
|  | **Higher** | 1.50 (1.11-2.02) | 1.32 (1.01-1.73) | 1.00 (ref) |
| ***By level of income*** | |  |  |  |
| **Education** | **Lower** | 0.87 (0.66-1.14) | 0.88 (0.69-1.13) | 0.90 (0.69-1.16) |
|  | **Medium** | 0.90 (0.68-1.19) | 0.89 (0.70-1.14) | 0.93 (0.75-1.15) |
|  | **Higher** | 1.00 (ref) | 1.00 (ref) | 1.00 (ref) |

P-value for interaction: 0.998

* Adjusted for all covariates listed in Table 1 except stroke

## Supplement Table 19. Hazard ratios with 95% CI for the association between socioeconomic factors and incident stroke, by sex.

|  | **Women** | | | **Men** | | | **P value*** |
| --- | --- | --- | --- | --- | --- | --- | --- |
|  | **Age-adjusted** | **Model 1** | **Model 2** | **Age- adjusted** | **Model 1** | **Model 2** |  |
| **Income** |  |  |  |  |  |  | 0.97 |
| Lower | 1.51 (1.21-1.88) | 1.37 (1.09-1.71) | 1.34 (1.07-1.68) | 1.48 (1.29-1.70) | 1.42 (1.23-1.63) | 1.40 (1.21-1.61) |  |
| Medium | 1.35 (1.07-1.70) | 1.26 (0.99-1.59) | 1.24 (0.98-1.57) | 1.34 (1.16-1.54) | 1.30 (1.12-1.50) | 1.28 (1.11-1.48) |  |
| Higher | 1.00 (ref) | 1.00 (ref) | 1.00 (ref) | 1.00 (ref) | 1.00 (ref) | 1.00 (ref) |  |
| **Education** |  |  |  |  |  |  | 0.18 |
| Lower | 0.96 (0.77-1.19) | 0.89 (0.72-1.12) | 0.87 (0.70-1.09) | 1.09 (0.92-1.28) | 1.04 (0.89-1.23) | 1.04 (0.88-1.22) |  |
| Medium | 0.83 (0.65-1.06) | 0.81 (0.63-1.03) | 0.80 (0.63-1.02) | 1.09 (0.93-1.28) | 1.06 (0.91-1.25) | 1.06 (0.90-1.24) |  |
| Higher | 1.00 (ref) | 1.00 (ref) | 1.00 (ref) | 1.00 (ref) | 1.00 (ref) | 1.00 (ref) |  |

Model 1: Adjusted for all covariates listed in Table 1 except outcome of interest, smoking and alcohol

Model 2: Adjusted for all covariates listed in Table 1 except outcome of interest.

*P-values for interaction by sex in fully-adjusted model

# Cardiovascular death

## Supplemental Table 20. Hazard ratios with 95% CI for cardiovascular death and interaction between income and education in the multivariable-adjusted* models.

|  |  | **Income** |  |  |
| --- | --- | --- | --- | --- |
|  |  | **Lower** | **Medium** | **Higher** |
| **Income** | **Lower** | 1.52 (1.37-1.68) | 1.47 (1.32-1.64) | 1.59 (1.34-1.90) |
|  | **Medium** | 1.34 (1.20-1.50) | 1.20 (1.07-1.35) | 1.26 (1.08-1.48) |
|  | **Higher** | 0.99 (0.86-1.15) | 0.91 (0.80-1.04) | 1.00 (ref) |
| ***By level of income*** | |  |  |  |
| **Education** | **Lower** | 0.97 (0.83-1.12) | 1.06 (0.92-1.22) | 0.93 (0.80-1.08) |
|  | **Medium** | 0.93 (0.80-1.09) | 0.95 (0.83-1.10) | 0.90 (0.79-1.03) |
|  | **Higher** | 1.00 (ref) | 1.00 (ref) | 1.00 (ref) |

P-value for interaction: 0.594

*Adjusted for all covariates listed in Table 1

## Supplement Table 21. Hazard ratios with 95% CI for the association between socioeconomic factors and cardiovascular death, by sex.

|  | **Women** | | | **Men** | | | **P value*** |
| --- | --- | --- | --- | --- | --- | --- | --- |
|  | **Age-adjusted** | **Model 1** | **Model 2** | **Age-adjusted** | **Model 1** | **Model 2** |  |
| **Income** |  |  |  |  |  |  | 0.50 |
| Lower | 1.57 (1.38-1.78) | 1.43 (1.26-1.63) | 1.42 (1.25-1.62) | 1.85 (1.71-2.00) | 1.66 (1.53-1.80) | 1.62 (1.50-1.76) |  |
| Medium | 1.34 (1.17-1.54) | 1.24 (1.08-1.43) | 1.23 (1.07-1.41) | 1.44 (1.32-1.57) | 1.37 (1.26-1.49) | 1.35 (1.24-1.47) |  |
| Higher | 1.00 (ref) | 1.00 (ref) | 1.00 (ref) | 1.00 (ref) | 1.00 (ref) | 1.00 (ref) |  |
| **Education** |  |  |  |  |  |  | 0.68 |
| Lower | 1.32 (1.15-1.51) | 1.21 (1.05-1.39) | 1.20 (1.04-1.38) | 1.30 (1.19-1.41) | 1.19 (1.09-1.30) | 1.18 (1.08-1.29) |  |
| Medium | 1.07 (0.92-1.25) | 1.05 (0.90-1.22) | 1.03 (0.89-1.22) | 1.12 (1.03-1.23) | 1.07 (0.98-1.17) | 1.06 (0.97-1.16) |  |
| Higher | 1.00 (ref) | 1.00 (ref) | 1.00 (ref) | 1.00 (ref) | 1.00 (ref) | 1.00 (ref) |  |

Model 1: Adjusted for all covariates listed in Table 1 except outcome of interest, smoking and alcohol

Model 2: Adjusted for all covariates listed in Table 1 except outcome of interest.

*P-values for interaction by sex in fully-adjusted model

# Figures

## Supplemental Figure 1. Smoothed multivariable-adjusted hazard functions according to family income and education for each outcome of interest.


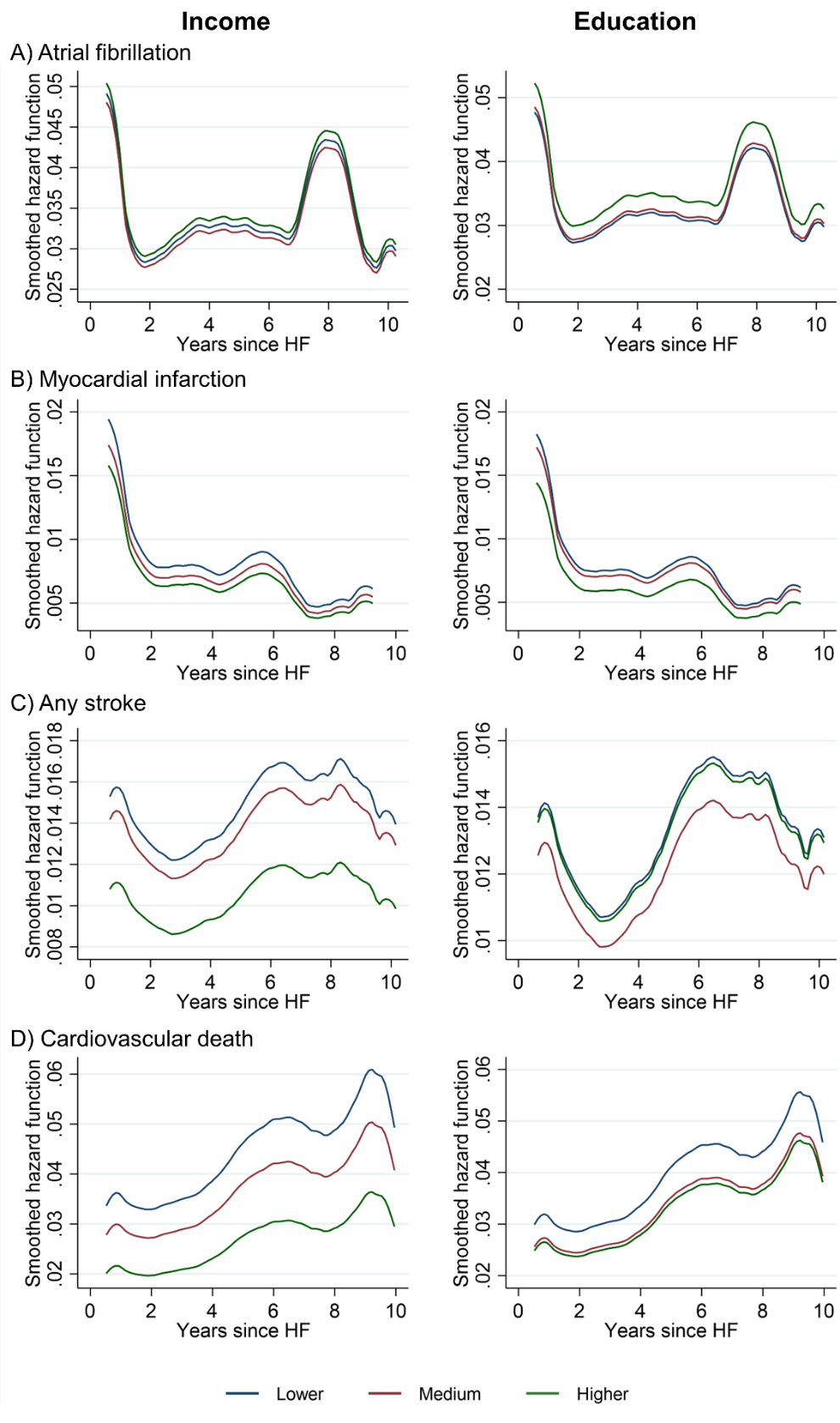


Cause-specific hazards correspond to the instantaneous rates of occurrence of the event of interest in subjects who are currently alive and event free. Estimated hazard functions are adjusted as in Model 3. Patients with missing information on education and/or income were excluded.
